# *PSEN1/SLC20A2* double mutation causes early-onset Alzheimer’s disease and primary familial brain calcification co-morbidity

**DOI:** 10.1007/s10048-023-00723-x

**Published:** 2023-06-21

**Authors:** Sophie Hebestreit, Janine Schwahn, Vesile Sandikci, Mate E. Maros, Ivan Valkadinov, Rüstem Yilmaz, Lukas Eckrich, Seyed Babak Loghmani, Hendrik Lesch, Julian Conrad, Holger Wenz, Anne Ebert, David Brenner, Jochen H. Weishaupt

**Affiliations:** 1 Division of Neurodegenerative Disorders, Department of Neurology, Mannheim Center for Translational Neurosciences, Medical Faculty Mannheim, Heidelberg University, Theodor-Kutzer-Ufer 1-3, 68167 Mannheim, Germany; 2 Department of Neurology, Medical Faculty Mannheim, Heidelberg University, Mannheim, Germany; 3 Department of Neuroradiology, Medical Faculty Mannheim, Heidelberg University, Mannheim, Germany; 4 Department of Neurology, University of Ulm, Ulm, Germany; 5 Department of Biomedical Informatics, Medical Faculty Mannheim, Heidelberg University, Mannheim, Germany

**Keywords:** Primary familial brain calcification, Alzheimer’s disease, Dementia, Cognitive disorder, Movement disorder, Neurogenetics, Neuroscience

## Abstract

Primary familial brain calcification (PFBC; formerly Fahr’s disease) and early-onset Alzheimer’s disease (EOAD) may share partially overlapping pathogenic principles. Although the heterozygous *loss-of-function* mutation c.1523 + 1G > T in the PFBC-linked gene *SLC20A2* was detected in a patient with asymmetric tremor, early-onset dementia, and brain calcifications, CSF β-amyloid parameters and FBB-PET suggested cortical β-amyloid pathology. Genetic re-analysis of exome sequences revealed the probably pathogenic missense mutation c.235G > A/p.A79T in *PSEN1*. The *SLC20A2* mutation segregated with mild calcifications in two children younger than 30 years. We thus describe the stochastically extremely unlikely co-morbidity of genetic PFBC and genetic EOAD. The clinical syndromes pointed to additive rather than synergistic effects of the two mutations. MRI data revealed the formation of PFBC calcifications decades before the probable onset of the disease. Our report furthermore exemplifies the value of neuropsychology and amyloid PET for differential diagnosis.

**Supplementary information:**

The online version contains supplementary material available at 10.1007/s10048-023-00723-x.

## Introduction

Primary familial brain calcification (PFBC; formerly Fahr’s disease) is a neuropsychiatric disorder that can manifest with movement disorders as well as cognitive and psychiatric symptoms. Mutations in several genes, including *SLC20A2*, *PDGFB*, *PDGFRB*, *XPR1*, or *MYORG*, cause autosomal-dominantly or -recessively inherited PFBC [1] with symmetrical perivascular calcifications in the basal ganglia, thalamus, cerebellum, and cortical and subcortical areas [2]. Although usually less pronounced, similar calcifications can also be observed in aged individuals without obvious symptoms or patients with neurodegenerative diseases such as Alzheimer’s disease [3, 4]. However, the possible pathogenic overlap between PFBC and tissue calcification of alternative origins as well as the possible role of mineral homoeostasis for protein aggregation in general [5] remains unresolved. In contrast to late-onset Alzheimer’s disease, early-onset Alzheimer’s disease (EOAD) is often caused by autosomal-dominant mutations, specifically in *APP*, *PSEN1*, and *PSEN2* [6]. Co-occurrence of mutations in genes causing PFBC and EOAD in the same patient could help estimating a potential cross-interaction between brain calcification and neurodegeneration, but has never been observed before.

## Methods

The index patient was referred to the outpatient clinic because of early-onset dementia with brain calcifications. The patient and his family members were recruited to the PFBC register “Fahr-NET” upon informed consent (see also “Declarations” section below). Whole exome sequencing and targeted Sanger sequencing was performed as described before [7]. Respective sequencing primers are available upon request. The detailed clinical work-up of the index patient included cerebral computed tomography (CT) and magnetic resonance imaging (MRI), cerebrospinal fluid (CSF) analysis with β-amyloid levels and β-amyloid 42/40 ratio, fluorbetaben-PET (FBB-PET), fluorodeoxyglucose-PET (FDG-PET), CSF analysis, neurological examination, and serial neuropsychological assessment (MOCA, CERAD).

## Results

Memory deficits and spatial disorientation of the index patient were first noted at the age of 53 years. At the age of 57 years, he presented for the first time to our clinic with spatial and temporal disorientation, forgetfulness, reduced motivation, disinhibition, and socially inadequate behavior. The basic neurological examination was normal. 21/30 points were achieved in the MMSE. Further neuropsychological testing revealed prominent deficits in episodic memory as well as executive deficits concerning planning, conceptualization, abstract thinking, flexibility, and monitoring. He showed disinhibited behavior and anosodiaphoria. In the following 3 years, all deficits progressed further with addition of visuoconstructive deficits and apraxia as well as reduced drive and attentional deficits.

**Fig. 1:**
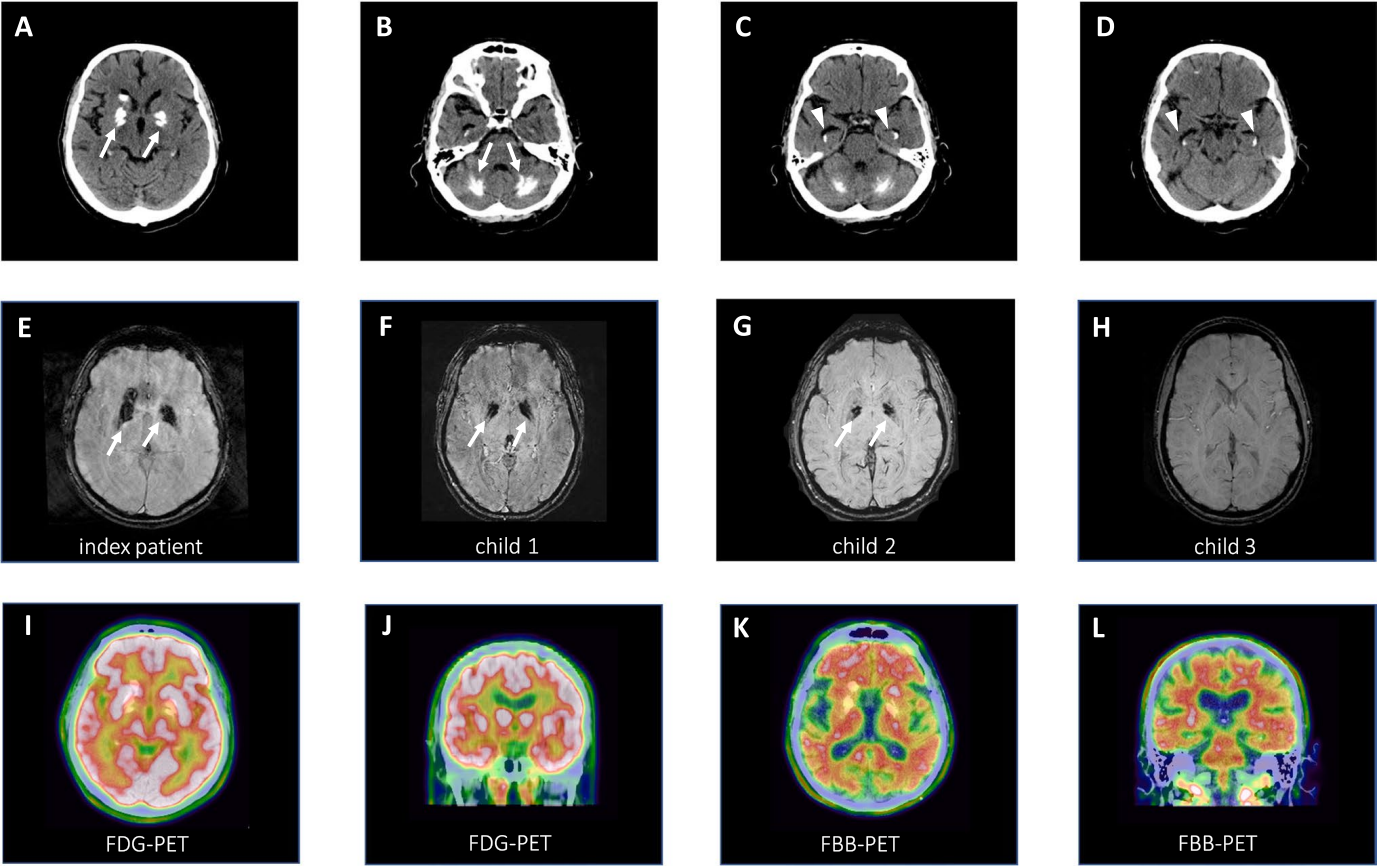
Axial cerebral CT and susceptibility-weighted MR imaging of the index patient (**A**–**E**) shows extensive calcifications of bilateral basal ganglia (in pallidum and caudate nucleus) as well as in both cerebellar hemispheres (arrows) and bilateral hippocampus (arrowheads). Susceptibility-weighted MR imaging of the index patient’s children (**F**–**H**) shows symmetrical calcifications of the basal ganglia (arrows) in both child 1 and child 2. Child 3 shows no calcifications. Axial and coronal FDG-PET/CT (**I**, **J**) of the index patient demonstrates areas of slightly reduced glucose metabolism in parietal and mesiotemporal cortex. FBB-PET/CT (**K**, **L**) study in axial and coronal sections shows widespread β-amyloid deposits in the frontal and temporal cortex but also in the parietal cortex. Red/light red indicates high values; green/blue indicates low values

CT and MR imaging revealed symmetrical calcifications in the pallidum, thalamus, cerebellum, and also the hippocampus (Fig. 1A–E), suggestive of PFBC. Whole exome sequencing and targeted analysis of known PFBC genes followed by Sanger sequencing confirmation (Suppl. Figure 1) revealed a heterozygous variant in *SLC20A2* (c.1523 + 1G > T), which is predicted to disrupt a splice donor site and lead to a translational frameshift. RT-PCR and Sanger sequencing of the PCR product was successfully used to detect wild-type but failed to identify mutant *SLC20A2* mRNA from patient whole blood (data not shown). This observation could be explained by the generally low expression of *SLC20A2* in blood cells ([8]; www.proteinatlas.org/ENSG00000168575-SLC20A2/single+cell+type) possibly together with nonsense-mediated RNA decay of the mutant mRNA. Taking together the symmetric brain calcifications and the fact that loss-of-function mutations in *SLC20A2* have repeatedly been shown to cause PFBC, it is highly likely that the *SLC20A2* variant is causative. Causality was further corroborated by segregation of the mutation with milder pallidal calcifications in two children of the patient, which were absent in a third child who was tested negative for the mutation (Fig. 1F–H). All three children were of full age but younger than 30 years. One of the two children carrying the *SLC20A2* mutation (Fig. 1F) suffers from panic disorder and depression but has no movement disorder or cognitive deficits (29/30 points in the MMSE). The other *SLC20A2* mutation carrier (Fig. 1G) is asymptomatic and achieved also 29/30 points in the MMSE. The third child, which was tested negative for the mutation (Fig. 1H), suffers from depression and 30/30 points were scored on the MMSE.

Secondary basal ganglia calcifications were excluded in the index patient. CSF analysis revealed normal values for total protein, CSF/serum albumin ratio, cell count, and lactate; there was no intrathecal IgG synthesis. In contrast, CSF tau, phospho-tau, and β-amyloid protein levels were compatible with Alzheimer’s disease. Remarkably, at the age of 57 years, i.e., 4 years after onset of symptom, CSF Tau protein and β-amyloid values had still been in the normal range (total tau protein 272 pg/ml (normal range < 445 pg/ml); β-amyloid 381 pg/ml (> 375 pg/ml)) or only marginally elevated (phospho-tau 68 pg/ml (< 61 pg/ml)). At 60 years of age, however, all markers were pathological (total tau protein 777 pg/ml (normal range < 400 pg/ml); phospho-tau 107 pg/ml (< 60 pg/ml); β-amyloid 1–42 359 pg/ml (> 600 pg/ml); β-amyloid 42/40 ratio 0.05 (> 0.07)). Consequent FDG-PET imaging showed a mildly reduced glucose metabolism in parietal and mesiotemporal cortical regions **(**Fig. 1I, J). Moreover, a FBB-PET study demonstrated widespread cortical β-amyloid deposits in cortical regions (Fig. 1K, L). Considering the CSF analysis and PET imaging results that were in agreement with Alzheimer’s disease pathology, re-analysis of the whole exome data was performed and revealed the genetic missense variant c.235G > A/p.A79T in the EOAD gene *PSEN1* that was confirmed by Sanger sequencing (Suppl. Figure 1). The variant alters a highly conserved amino acid and is predicted to be damaging (CADD score = 33; predicted to be “probably damaging” by polyphen2). The index patient’s children were all tested negative for the mutation.

The rarity of this variant in reference databases (gnomAD allele frequency 4/251,404) and the fact that another missense mutation in the same codon has been observed before in three other families affected by EOAD [9] further corroborates its causality, and amino acid 79 of PSEN1 could represent a mutational hotspot. The *APOE* genotype was found to be *ɛ3* (homozygous reference sequence) in this patient. The mother of the patient has been reported to be “demented,” but paper prints of her cranial CT shown to the authors ruled out calcifications. It is therefore likely that the *PSEN1* mutation was transmitted from the index patient’s mother but the *SLC20A2* mutation from the father.

## Discussion

We present the first patient with comorbid PFBC and genetic EOAD. Since the estimation of the population frequency of PFBC is up to 2.1:1000 [10] and the frequency of genetic EOAD is approximately 5:100.000 (prevalence EOAD ca. 25–50:100,000; approximately 10% of EOAD are caused by Mendelian mutations [6]), the likelihood of a PFBC and EOAD double mutation can be roughly estimated as 1:9,5 Mio.

Vascular risk factors are generally associated with AD, and AD is often accompanied by vascular deposition of β-amyloid (cerebral amyloid angiopathy; CAA) that influences the manifestation of AD [11]. Similarly, vascular β-amyloid pathology has been described before in PFBC [12], which is usually characterized by mainly perivascular calcifications. Moreover, all known PFBC disease genes are predominantly expressed in cells forming the blood brain barrier (astrocytes, endothelial cells, pericytes) [13]. Thus, the vascular involvement in both AD and PFBC could be the correlate of partially shared pathogenic mechanisms. The role of mineral homoeostasis for aggregation of disease-linked molecules has also been debated for a long time [5]. Surprisingly, however, an obviously synergistic mutual interaction of neurodegeneration and brain calcification was not observed in the index patient. We cannot prove a strictly independent co-occurrence of genetic PFBC and EOAD in the case presented. Both mutations could theoretically represent low-effect size variants acting together to cause the reported syndrome, although loss-of-function mutations in *SLC20A2* usually have a high penetrance. Moreover, alteration of tissue homoeostasis and disturbance of the blood brain barrier due to PFBC could have preponed EOAD, and the observed hippocampal calcifications may be the combined result of Alzheimer’s disease pathology and a genetic predisposition to brain calcifications due to the *SLC20A2* mutation. Nevertheless, dementia markers that remained normal for several years after onset as well as moderate cognitive and behavioral symptoms 7 years after disease onset despite the usually faster progressive course of EOAD [6] argue against an exceptionally aggressive disease course resulting from a synergistic action of the two mutations. In line with this view, no overlap in gene ontology terms was found for *PSEN1* and *SLC20A2* (data not shown).

Thus, despite the possibly overlapping pathogenic mechanisms of PFBC and AD based on a shared vascular involvement or a possible role of mineral dys-homoeostasis for protein aggregation, the stochastically extremely unlikely double mutation reported here nevertheless suggests mechanistically differential rather than direct synergistic contributions of interacting pathomechanisms in the two diseases. Moreover, the described clinical work-up underscores the relevance and value of both a detailed neuropsychological examination and amyloid-PET imaging for the differential diagnosis of dementia. While neuropsychological diagnostics led to the otherwise unlikely working hypothesis of Alzheimer’s disease co-morbidity in an PFBC patient, the latter could confirm the surprising finding of amyloid pathology in an individual primarily diagnosed with PFBC. Finally, our MRI-based detection of basal ganglia calcification in two young *SLC20A2* mutation carriers suggests a slow, linearly progressing formation of PFBC-linked calcifications starting decades before symptom onset.

## Supplementary information

Below is the link to the electronic supplementary material.

Supplementary file1 (DOCX 1956 KB)

## Data Availability

Sequencing raw data and imaging data/MRI data are available upon request.
