# Supplementary material for: PSEN1/SLC20A2 double mutation causes early-onset Alzheimer’s disease and primary familial brain calcification co-morbidity
Source: Neurogenetics. 2023 Jun 21;24(3):209–13. doi: 10.1007/s10048-023-00723-x (PMC10319679; doi:10.1007/s10048-023-00723-x)
Supplement: Supplementary file 1 — Supplementary file1 (DOCX 1956 KB) [file 10048_2023_723_MOESM1_ESM.docx]

**Supp. figure 1:** Sanger sequencing of the index patient and his three children showing the A) *SLC20A2* (c.1523+1G>T) and B) *PSEN1* (c.235G>A) variants.

**A.**

**Exon 8**


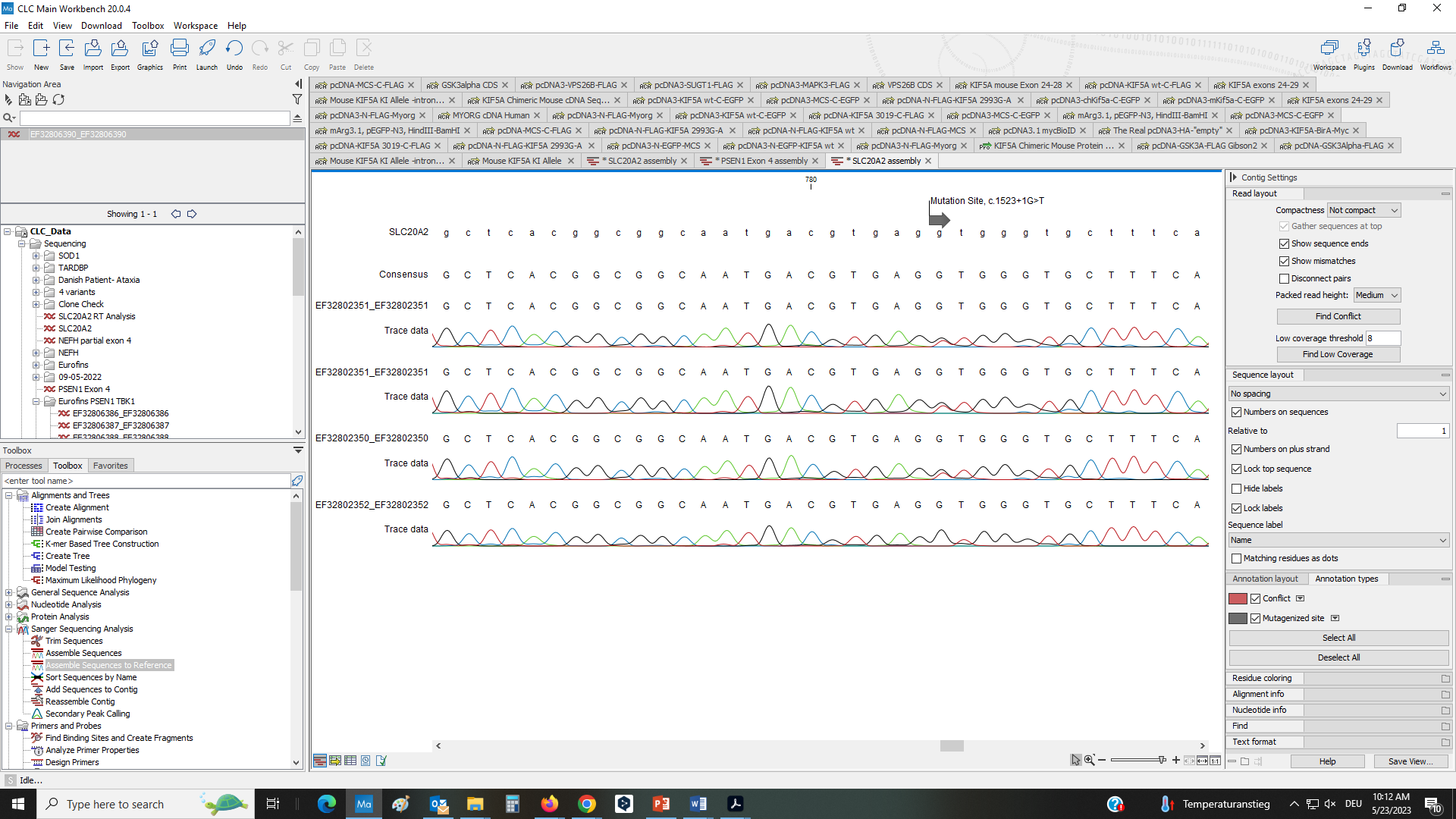

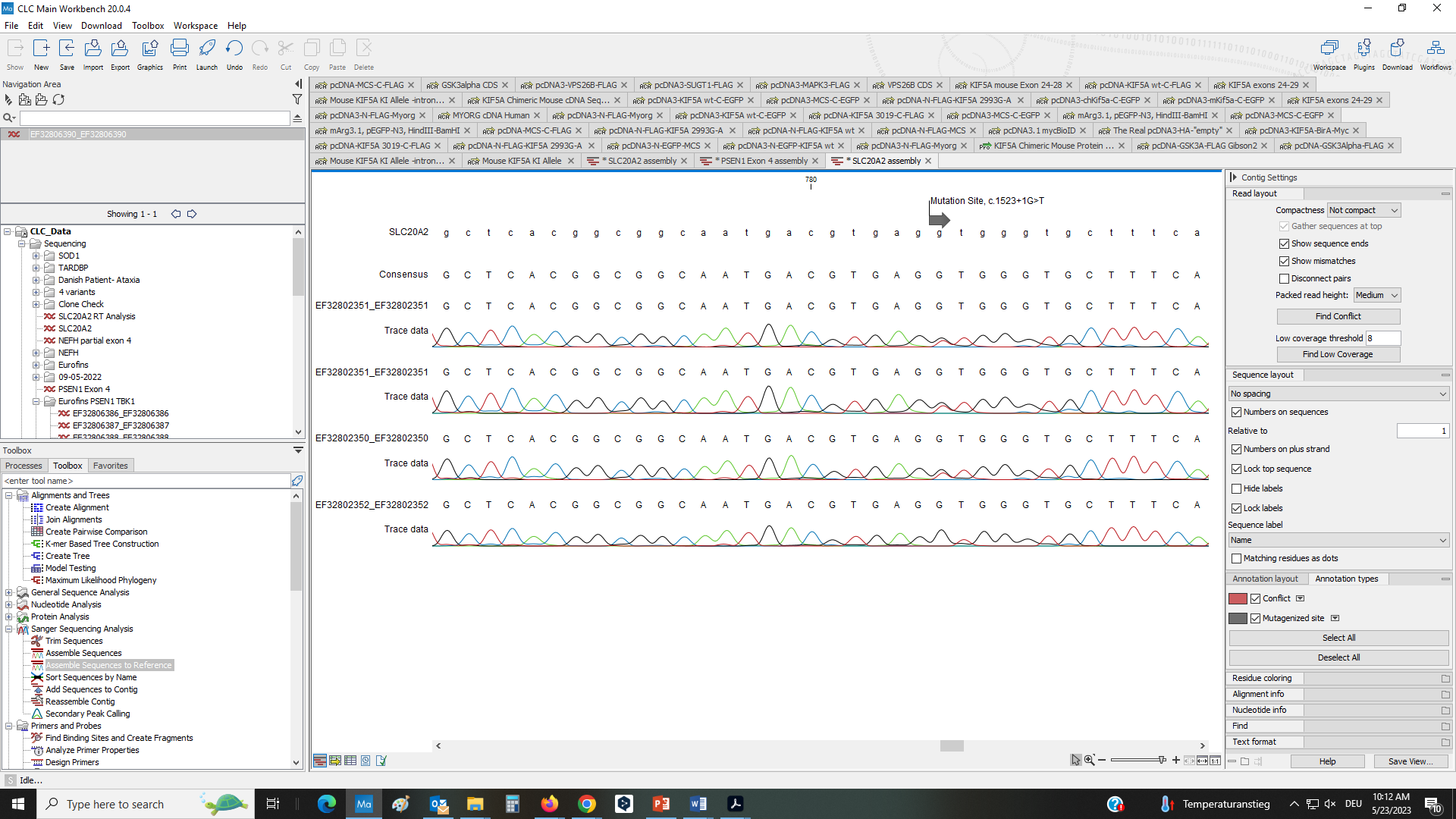

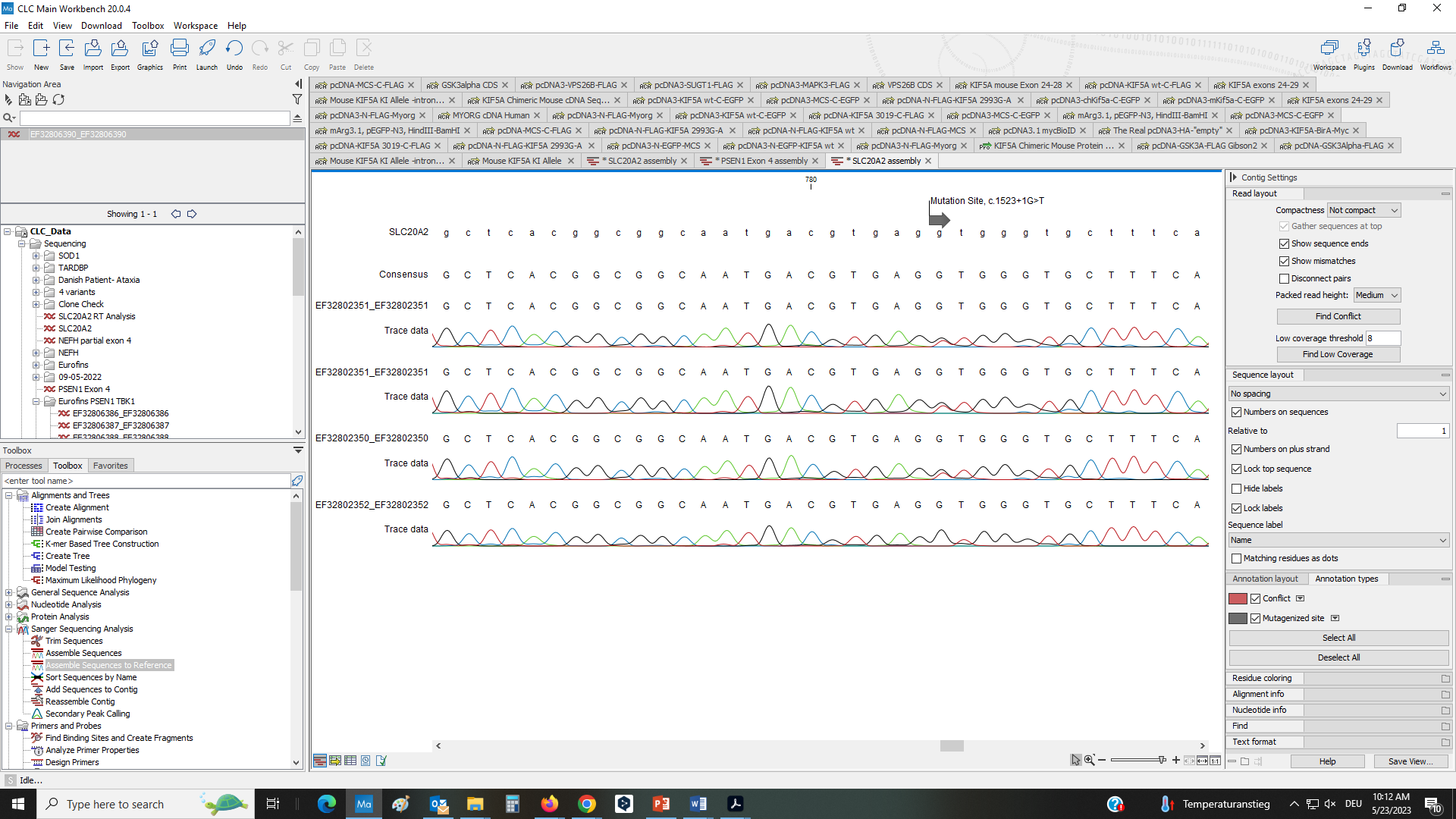


**Reference**

**Index patient**

**Child 1**

**Child 2**

**Child 3**

**B.**

**Exon 4**

**Reference**


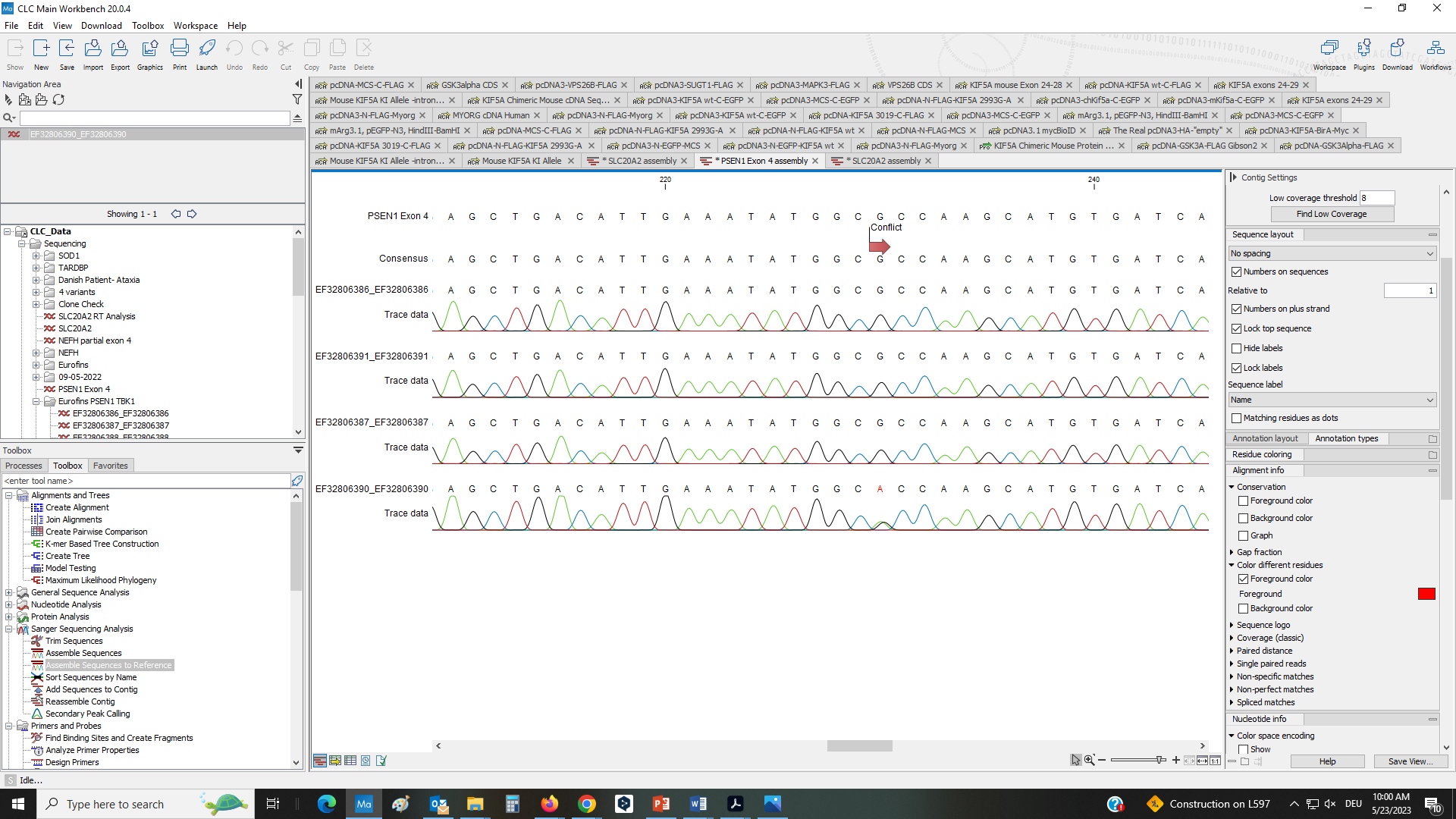

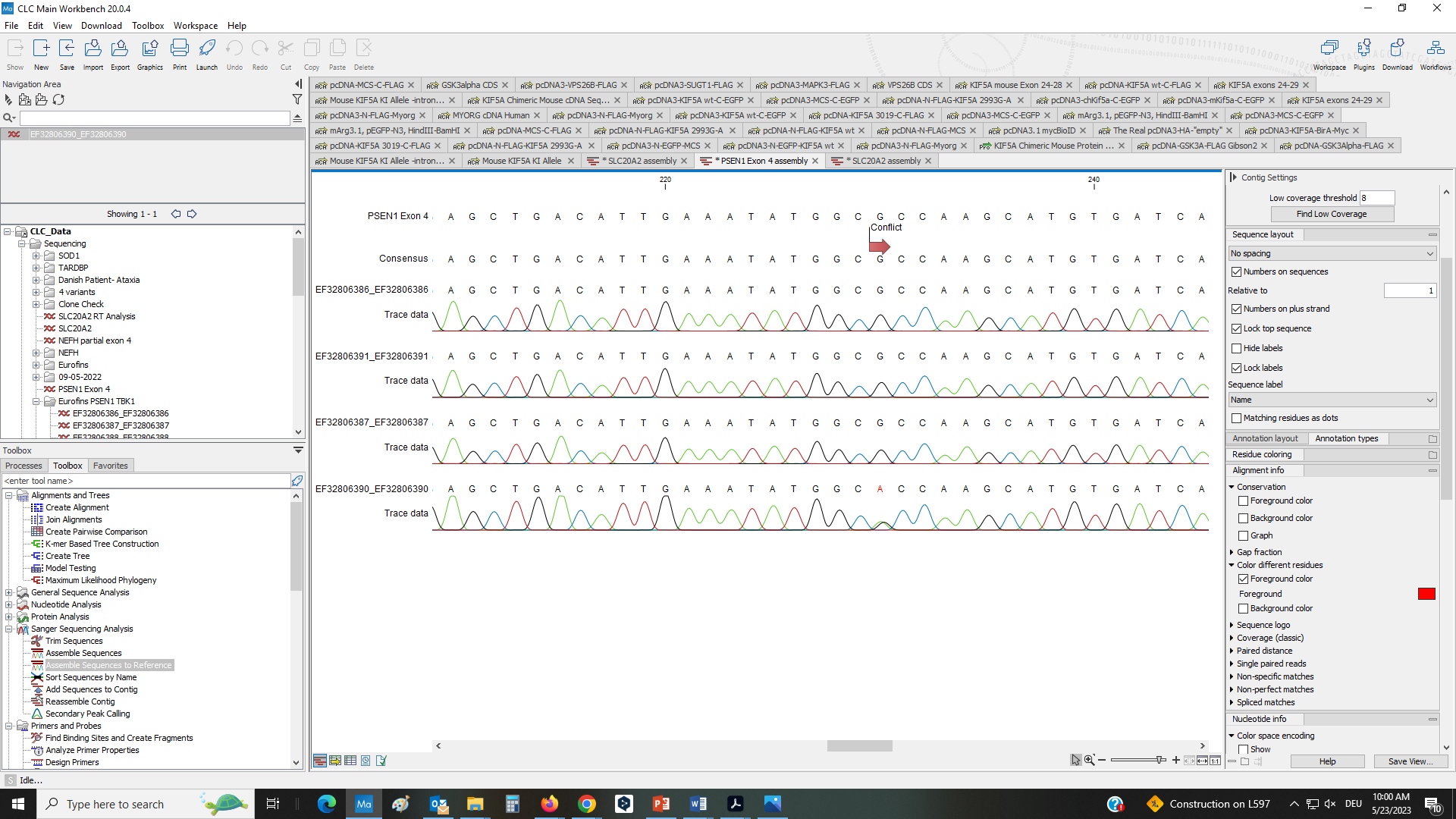

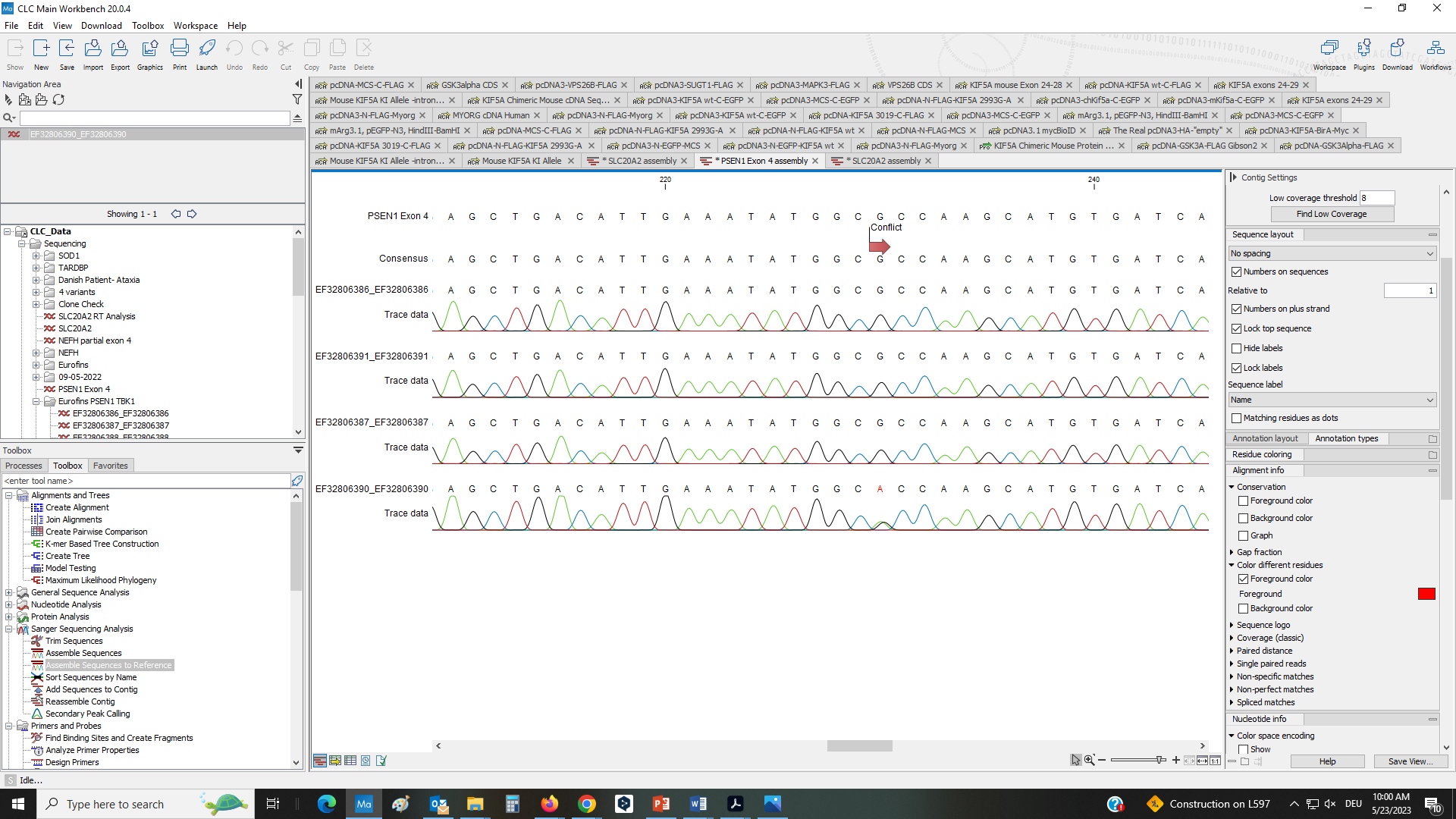


**Child 3**

**Child 2**

**Child 1**

**Index patient**
